# Human Endometrium Derived Induced Pluripotent Stem Cells Are Amenable to Directed Erythroid Differentiation

**DOI:** 10.1007/s13770-023-00554-9

**Published:** 2023-07-15

**Authors:** Hyun Kyung Kim, SiHyun Cho, Young Sik Choi, Byung Seok Lee, Sinyoung Kim, Hyun Ok Kim, Joo Hyun Park

**Affiliations:** 1 Department of Obstetrics and Gynecology, Yongin Severance Hospital, Yonsei University College of Medicine, 363, Dongbaekjukjeon-Daero, Giheung, Yongin, 16995 Gyeonggi-Do Republic of Korea; 2 Department of Obstetrics and Gynecology, Gangnam Severance Hospital, Yonsei University College of Medicine, Seoul, 06273 Republic of Korea; 3 Institute of Women’s Life Medical Science, Yonsei University College of Medicine, Seoul, 03722 Republic of Korea; 4 Department of Obstetrics and Gynecology, Severance Hospital, Yonsei University College of Medicine, Seoul, 03722 Republic of Korea; 5 Department of Laboratory Medicine, Yonsei University College of Medicine, Seoul, 03722 Republic of Korea

**Keywords:** Induced pluripotent stem cells, Hematopoietic stem cells, Erythroid differentiation, Endometrium, Regenerative medicine

## Abstract

**BACKGROUND:**

A protocol for using human endometrium derived induced pluripotent stem cells (iPSCs) to derive hematopoietic and erythroid lineages will be elaborated, through a two-phase culture system.

**METHODS:**

Discarded endometrial tissues were obtained from women receiving hysterectomy in their 4th to 5th decade due to benign uterine conditions. pCE-Sox2, Oct4, Klf4, L-Myc and Lin28 episomal vectors were used to electrotransfect the endometrial stromal cells. The first 8 days involves commitment to hematopoietic stem cells through embryoid body with robust expansion on murine bone marrow stromal cells. The second phase involves feeder free conditions with hydrocortisone, stem cell factor, interleukin-3, and recombinant EPO. After 22 days of feeder free culture, the expression profiles of CD235a^+^, CD34^+^, CD43^+^ and CD 71^+^ were analyzed by flow cytometry and Wright-Giemsa staining for differential counting. The oxygen carrying capacity of cultured RBCs was measured using a hemoxanalyser.

**RESULTS:**

As a result of inducing these cells via co-culture with murine stromal fibroblasts, all endometrium derived iPSCs were differentiated into erythroblasts with a stable yield of approximately 80% for polychromatic and orthochromatic normoblasts. The protocol for complete induction of erythroid lineage cells starting from human endometrial tissue via iPS cells has been optimized.

**CONCLUSION:**

Successful directed erythroid differentiation has occurred from human endometrium-derived iPS cells. A comprehensive process of actually deriving iPS cells using discarded surgical hysterectomy specimens to the erythroid fate has significance in that the scope of using human iPSC cell lines for tissue regeneration could be expanded in the future.

**Supplementary Information:**

The online version contains supplementary material available at 10.1007/s13770-023-00554-9.

## Introduction

In the field of obstetrics and gynecology, transfusion of red blood cells is a common practice, and discovering a safer and readily available source of transfusion for acute and chronic blood loss could significantly improve patient care. Anemia may not always be adequately corrected conservatively by means of increased dietary intake or iron supplementation, and transfusion usually offers immediate replacement for acute or chronic blood loss. However, transfusion has its own disadvantages and finding an alternative, preferably an autologous source of transfusion, is the need of the hour. Transfusion of red blood cells rely mainly on donation where supply may be unstable, affected by epidemic outbreaks of infectious diseases, and also rare events including anaphylactic reaction, infectious disease transmission and alloimmunization may occur, which could be fatal [1].

The human endometrium is an easily replenished and accessible tissue that is clinically obtainable by means of endometrial curettage and alternatively as discarded tissue after hysterectomy procedures. Due to its abundance, regenerative potential, and plasticity, endometrial tissue has been previously described in literature as a favorable source for tissue regeneration and engineering [2, 3]. From the basalis layer, the endometrial gland and stroma expand quantitatively and undergo qualitative cellular transformation within a window of 3–4 weeks in order to become receptive to the fertilized embryo. Human endometrium-derived induced pluripotent stem cells (hEm-iPSCs) have been previously described as an efficient cell source for reprogramming into a pluripotent state [3, 4]. In addition, resident adult stem cells in the basalis layer of the endometrium have been proposed as a source for *in vitro* differentiation into various lineages, owing to their high plasticity [2].

Induced pluripotent stem cells (iPSCs), first described in 2006 using murine fibroblasts, have opened a new avenue in regenerative medicine [5]. The ethical dilemma faced with embryonic stem cells (ESCs), which involves retrieval of the inner cell mass arising from fertilized embryos, is not encountered with iPSCs. iPSCs are generated by overexpressing fully differentiated somatic cells with ectopic factors involved in the transcriptional regulation of early embryogenesis or cell cycle regulation to induce dedifferentiation into a state of pluripotency similar to ESCs. These factors were first defined in 2006, using retroviral vector transduction with murine fibroblasts, which included Sox2, Oct4, Klf4, and c-Myc [5]. Subsequently, human iPSCs were established using lentiviral Sox2, Oct4, Nanog, and Lin28 [6]. As iPSCs were investigated further, various forms of transfection or transduction modalities were developed in order to minimize ectopic gene integration and enhance reprogramming efficiency, including Sendai virus [7–9], proteins [10], episomal vector [11, 12], mRNA [8] and miRNA [13].

Hematopoietic stem cells (HSCs) arise from the mesodermal precursor cell lineage and the specific cell surface markers characterizing each stage of erythrocyte differentiation are presently known [14, 15]. In the early stage of differentiation, the endothelial precursor populations CD34^+^ (transmembrane phosphoglycoprotein) and CD43^+^ (leukosialin), which upregulate CD235a^+^ (glycophorin A), are expressed. In the later stages, CD43^+^, CD235a^+^, and CD71^+^ are concomitantly expressed [16–19]. Multipotent progenitors originating from HSCs subsequently give rise to erythroid progenitors (erythroblasts; EBLs) and megakaryotic progenitors [20]. Based on the specific marker expression patterns occurring during *in vivo* hematopoiesis, hallmark markers for *in vitro* differentiation are selected in a stepwise manner. Vascular endothelial growth factor receptor 2 precursor (VEGFR2 or KDR) is an indicator of mesodermal commitment prior to hematopoietic differentiation. During the process of hematopoietic differentiation, the expression of CD34^+^CD43^+^ increases and as commitment to the common myeloid progenitor becomes evident, CD235a^+^CD71^+^ expression is known to increase, whereas the CD34^+^CD43^+^ cell population slowly decreases. The proportion of CD235a^+^ is expected to increase as erythroblast maturation proceeds [21, 22].

In this study, endometrial cell-derived iPSCs using postoperative discarded endometrium, were utilized to successfully differentiate and expand erythroblasts. Using a source which may be replenished and stored indefinitely, developing a novel source of transfusable RBCs could be made possible.

## Materials and methods

### Study design and cell sources

After receiving informed consent, five patients undergoing benign hysterectomies were enrolled in this study under the authorization of the Institutional Review Board of Severance Hospital (IRB No. 9-2021-0040).

Human endometrial tissues were obtained from patients in their 4th or 5th decade, at the Department of Obstetrics and Gynecology, Yonsei University College of Medicine, Gangnam Severance Hospital, Seoul, Korea. After obtaining a full-thickness (15 × 5 mm) wedge from the hysterectomy specimen, excluding any grossly noticeable pathologic lesions, the endometrial layer was dissected under a dissecting microscope to remove any myometrial fragments to avoid contamination by other cell sources. After mincing the endometrium with a surgical blade (Swann-Morton, Sheffield, England), the fragments were treated with ACK lysis buffer (Lonza, Allendale, NJ, USA) at a concentration of 1 ml per 20 ml of tissue suspension.

The fragments were then thoroughly washed twice with PBS (Corning, Steuben County, NY, USA) and immersed in 2 ml of collagenase IV (Life Technologies, Gibco, Grand Island, NY, USA) at 37 °C in a 5% CO_2_ incubator (Thermo Fisher Scientific, OH, USA) for 90–120 min to release individual cells. The cells were carefully pipetted every 15–30 min via a wide-bore 1000 uL tip to hasten the lysis process. In cases where lysis appeared to be relatively slow, additional collagenase IV was added. The cells were then washed and plated within the basal endometrium media containing DMEM-high glucose (Life Technologies, Paisley, PA49RF, UK), 10% fetal bovine serum certified (Life Technologies, NY, USA), and 1% penicillin–streptomycin (Life technologies, USA), until almost confluent (90–95%). The primary cells were split at a ratio of 1: 3 for passaging and stored in liquid nitrogen at passage 3 for future hiPSC derivation [3].

### Establishment of iPSCs from endometrial cells

#### Reprograming of human endometrial cells

Human endometrial iPSCs were derived from endometrial cell cultures at passage 3–4. Primary human endometrial cells were reprogrammed to hEm-iPSCs using the Epi5™ Episomal iPSC Reprogramming Kit (Life Technologies, Frederick, MD, USA). Before transfection, each well in a 6-well plate (Thermo Fisher Scientific, Jiangsu, China) was coated with a mixture of 12.5 uL of Matrigel® Matrix (Corning, Kennebunk, ME, USA) and 987.5 uL of DMEM/F12 (Life Technologies, Grand Island, NY, USA) for 1 h at room temperature. The appropriate amount of Human endometrial cells (5 × 10^5^ cells) per one well of a 6 well plate which was determined by repeated experiments, was plated after counting with a hemocytometer using trypan blue dye and resuspended in 104 µL of media (18 µL of Supplement 1, 82 µL of Nucleofactor solution, both from P3 primary cell 4D-Nucleofactor kit, Lonza Amaxa, Germany), 2 µL of EpiTM episomal reprogramming vectors, namely pCE-hOCT3/4 (*Oct4*), pCE-hSK (*Sox2, Klf4*), pCE-hUL (*L-Myc*, *Lin28*), and 2 µL of Epi5™ p53 and EBNA vectors, namely pCE-mP53DD (mp53DD), pCXB-EBNA1 (EBNA1) (Life Technologies, Frederick, MD, USA). The prepared cells were transferred to a 100 µL Nucleocuvette ™ Vessel (Lonza, Koln, Germany) and loaded in the 4D-Nucleofector ™ System (Lonza, Koln, Germany). The electrotransfection under the “HeLa cell” human cell type program, was performed following the manufacturer’s instructions [23]. After removing the cuvette, processed cells were transferred into 3 wells containing 2 mL of endometrial medium at a density of 1.6 × 10^5^ cells/well in each well of a Matrigel pre-coated 6-well microplate. The cells were incubated in a 5% CO_2_ incubator at 37 °C.

To each well, 1 mL of endometrium media was added on post-transfection day 2 and 1 mL of ReproTeSR™ Basal Medium (Stem Cell Technologies, Vancouver, BC, Canada) on days 3 and 5. After 7 days, daily complete media change was performed with 2 mL of ReproTeSR™, and the plates were closely observed until colonies with an iPSC-like appearance were seen. Usually, iPSC-like colonies appear after 14 days. The colonies were manually picked based on their morphology between days 14 and 24 under a polarizing microscope, and cultured/passaged as iPSCs thereafter. iPSC line using healthy donor peripheral blood was also derived through the same process, which was used as control with matching racial background.

#### Detection of pluripotent germ layer markers in the established hEm-iPSCs

Third- or fourth-passage hEm-iPSC and H9 cells (NIH code WA09, WiCell Research Institute, Inc, Madison, Wisconsin), which are the most widely distributed and researched human ESC lines, were maintained on 10-cm plates coated with 0.1% gelatin solution (GenDEPOT, TX, USA) in 10 ml endometrium medium. The cells were grown for 2 weeks, while changing the medium once every 2–3 days. mRNA was extracted from the cells grown in this way to generate cDNA. Total RNA from endometrial cells and erythroblasts (14 days) was isolated using the RNeasy Plus Mini Kit (Qiagen, Tubingen, Germany). Complementary DNA was generated with the SuperScript III First Strand (Invitrogen, Thermo Scientific, CA, USA) and analyzed by quantitative real-time RT-PCR (qRT-PCR) using TaqMan Gene Expression Master Mix (Applied Biosystems, CA, USA) and Step One Plus (Applied Biosystems). The qPCR TaqMan probes (Applied Biosystems) used were as follows: Brachyury Hs00610080_m1_FAM; GSC Hs00418279_m1_FAM; MIXL1 Hs00430824_g1_FAM; Sox17 Hs00751752_s1_FAM; Nestin Hs04187831_g1_FAM; GAPDH Hs02758991_g1_VIC. *GAPDH* was used to normalize the data, and relative expression was calculated using the ΔΔC_T_ method. The significance of the difference between the samples was analyzed by t-test using Prism 9. **** *p* < 0.0001, *** *p* < 0.001, mean ± SE (*n* = 3).

#### Maintenance of human endometrium-derived iPSCs (hEm-iPSCs)

hEm-iPSC, hPB-iPSC (IRB No. 4–2018-0890) and H9 cells were maintained in 6-well plates coated with 10 ng/mL Vitronectin XF™ (Stem Cell Technologies, Vancouver, Canada) in 2 mL of mTeSR™ 1 media (Stem Cell Technologies, Vancouver, Canada). All cells were cultured at 37 °C under 5% CO_2_ and fed daily with mTeSR™ 1 media until the cells reached 70–80% confluence. Generally, the cell cultures grown this way are ready for passage within 4–7 days. hEm-iPSCs were enzymatically passaged in ReLeSR TM media.

### Differentiation into hematopoietic stem cells

#### Maintenance of OP9 cells

OP9 cells (murine embryonic mesenchymal stem cells, CRL-2749, ATCC, USA), a cell line derived from mouse bone marrow stromal cells, were plated on gelatinized (0.1% gelatin solution, GenDEPOT, TX, USA) 100-mm plates containing OP9 growth media (MEM alpha modification media, 1X, GE Healthcare Life Sciences, Utah, USA; 20% fetal bovine serum, Life Technologies, NY, USA). After the formation of confluent cultures on day 2, the medium was changed, and cells were cultured for an additional 2–3 days to allow for the expansion of the extracellular matrix.

#### Generation of embryoid bodies (EBs)

Prior to conducting directed hematopoietic differentiation, EB formation was optimized for both hEm-iPSCs, hPB-iPSCs and H9 cells, plated at 1 × 10^6^ cells on the AggreWell™ plate (Stem Cell Technologies, Vancouver, Canada) according to the manufacturer’s instructions [24]. On differentiation day 0, EBs were established using AggreWell^TM^400 plates (Stem Cell Technologies, Vancouver, Canada) according to the manufacturer’s instructions. AggreWell^TM^400 was pre-treated with 2 mL of AggreWell™ rinsing solution to remove any bubbles lodged within the wells (centrifugation at 1300 × g, 5 min). Single cells were derived from a monolayer culture of hEm-iPSC/H9 cells by enzymatic treatment with Easy Gentle Cell Dissociation Reagent (Stem Cell Technologies) at 37 °C for 5 min. Approximately 2 × 10^6^ cells were seeded into each well of the AggreWell™ plate with 5 mL of AggreWell™ EB Formation Medium (Stem Cell Technologies) containing 10 µM Y-27632 (Stem Cell Technologies) and 5 ng/mL bone morphogenic protein 4 (BMP4, Peprotech, NJ, USA). Cell aggregation was achieved by gentle centrifugation at 1300 × g for 5 min. Next day, half change of the EB Formation Medium containing 10 ng/mL BMP4, 6 ng/mL WNT3a (PeproTech), 6 ng/mL Activin-A (Peprotech), 5 ng/mL basic fibroblast growth factor (bFGF, Peprotech), and 5 ng/mL vascular endothelial growth factor (VEGF, PeproTech) was replaced. Two days later, EBs were harvested from microwells by firmly pipetting the medium in the well up and down 2–3 times with a micropipette outfitted with a 1-mL Axygen wide tip (Corning, NY, USA) to dislodge the EBs, and strained through a 40-cm cell strainer (Falcon, MA, USA).

#### Hematopoietic differentiation of EB/OP9 cocultured cells

The hematopoietic differentiation protocol was performed following the previously published protocol [18]. The EBs were added to overgrown OP9 cultures at a density of 7000 EBs (2 × 10^6^ cells) per 100-mm dish in 10 mL of differentiation medium (MEM alpha modification media, 10% fetal bovine serum, 100 μM/mL monothioglycerol; Sigma-Aldrich, MO, USA) containing 10 ng/mL BMP4, 6 ng/mL WNT3a, 6 ng/mL, 5 ng/mL bFGF, and 5 ng/mL VEGF. On the following day (day1), the entire medium was aspirated and replaced with 20 mL of differentiation medium containing only 5 ng/mL VEGF. The EB/OP9 cocultures were incubated for up to 8 days at 37 °C in a 5% CO_2_ incubator with a half-medium change on days 4 and 6. To collect cells on day 8, a single cell suspension was prepared by treatment of the EB/OP9 cocultures with 1 mg/mL collagenase IV for 30 min at 37 °C followed by treatment with 0.05% trypsin/EDTA for 15 min at 37 °C. Differentiation medium was then added to neutralize the cells.

### Differentiation of hematopoietic lineage cells into erythroblasts

**Fig. 1:**
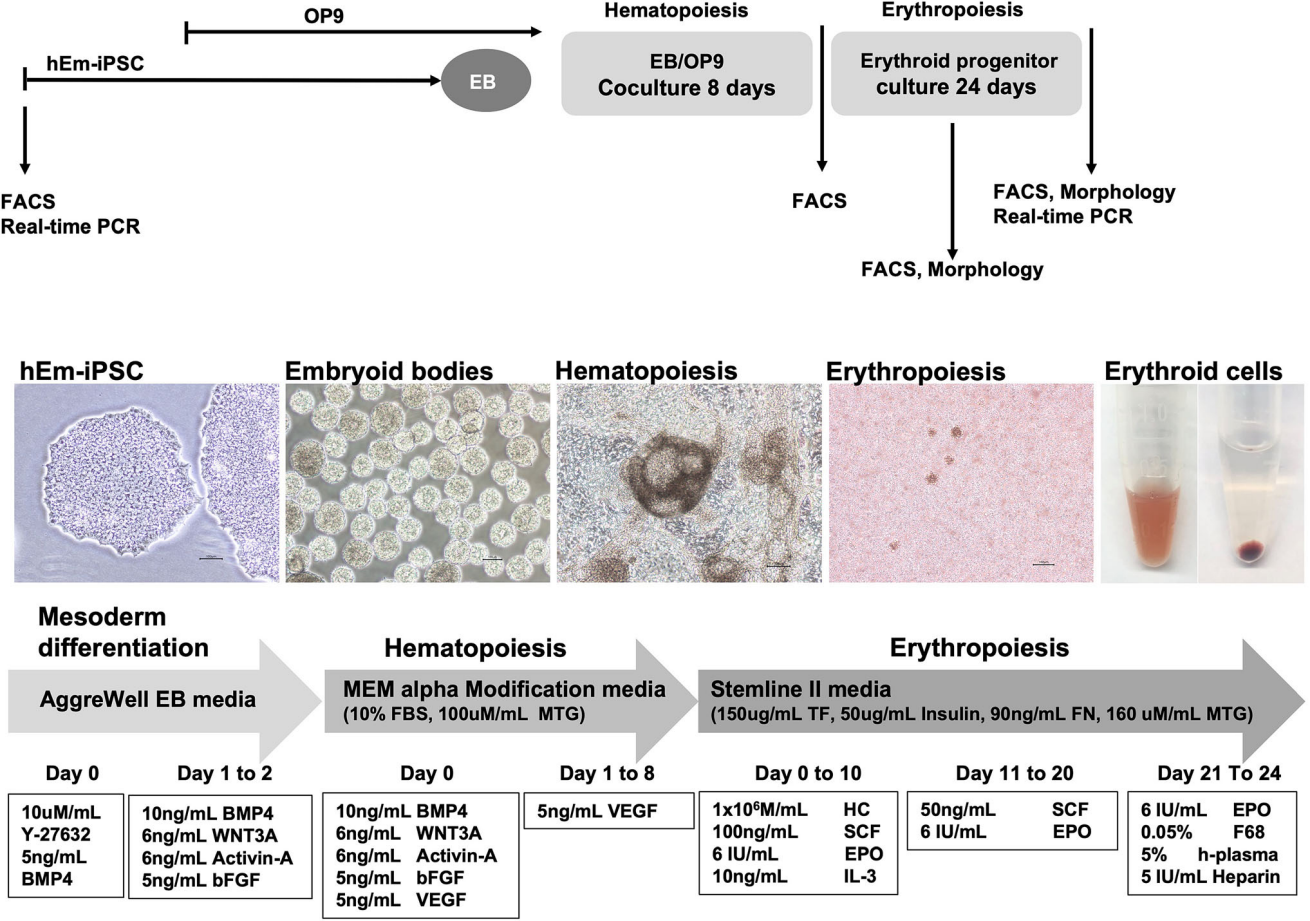
The time schedule of detection of differentiation into erythroid lineage and the scheme of the establishment protocol for complete induction of red blood cells starting from human endometrial iPSCs

The erythropoiesis protocol was performed following the previously published protocol [25, 26]. After 8 days of coculture, the EB/OP9 cells were replated as erythroid liquid culture. The cells were seeded at a density of 1 × 10^6^ cells/mL on a T25 plate (Thermo Fisher Scientific, Jiangu, China) containing basal media (Stemline II hematopoietic stem cell expansion medium, supplemented with 150 µg/mL iron-saturated human transferrin, 90 ng/mL ferric nitrate, 50 μg/mL insulin, 1 × 10^−6^ mol/L monothioglycerol, Sigma-Aldrich, MO, USA) with the following cytokines: 1 × 10^−6^ M/L hydrocortisone (Sigma-Aldrich), 100 ng/mL stem cell factor (SCF) (Peprotech, NJ, USA), 10 ng/mL interleukin-3 (IL-3) (Peprotech), and 6 IU/mL recombinant erythropoietin (EPO, JWpharma, Seoul, Korea). On days 11 to 20, the cells were replated every 3 days in basal media containing the following cytokines: 50 ng/mL SCF and 6 IU recombinant EPO. After 21–24 days, the cells were replated in basal media containing the following cytokines: 6 IU/mL recombinant EPO, 0.05% poloxamer 188 (Pluronic F 68, Sigma-Aldrich, MO, USA), 5% human plasma, and 5 IU/mL heparin (Stem Cell Technologies, Vancouver, Canada). All cultures were incubated at 37 °C in a humidified atmosphere containing 5% CO_2_. The experimental protocol for the *ex vivo* generation of hematopoietic and erythroid cells from hEm-iPSC lines is shown in (Fig. 1).

### Flow cytometric analyses of erythroid markers

To verify that directed hematopoietic differentiation occurred in the appropriate direction, flow cytometry was conducted. Hematopoietic and erythroid differentiated cells were stained with anti-mouse IgG-FITC, IgM-PE, and IgG-APC; PE-mouse anti-human CD235a, and CD34; and APC mouse anti-human CD71 and CD43 (BD Biosciences, CA, USA), for 30 min at 4 °C. The cells were washed and resuspended in magnetic activated cell sorting (MACS) buffer (Milltenyi Biotec, Bergisch Gladbach, Germany) and 4% paraformaldehyde (Tech & Innovation, Seoul, Korea) for cell data acquisition using a BD FACS Aria III flow cytometer (BD Biosciences, CA, USA). Data analysis was performed using the FlowJo software (BD Biosciences, CA, USA).

### Quantitative real-time polymerase chain reaction

Total RNA from endometrial cells and erythroblasts (14 days) was isolated using the RNeasy Plus Mini Kit (Qiagen, Tubingen, Germany). Complementary DNA was generated with the SuperScript III First Strand (Invitrogen, Thermo Scientific, NY, USA) and analyzed by qRT-PCR using TaqMan Gene Expression Master Mix (Applied Biosystems, Vilnius, Lithuania) and Step One Plus (Applied Biosystems, CA, USA). The qPCR TaqMan probes (Applied Biosystems, CA, USA) used were as follows: GATA1 Hs0185823_m1_FAM; GATA2 Hs00231119_m1_FAM; TAL1 Hs01097987_m1_FAM; KLF1 Hs00610592_m1_FAM; GAPDH Hs02758991_g1_VIC. *GAPDH* was used to normalize the data, and relative expression was calculated using the ΔΔC_T_ method. The significance of the difference between samples was analyzed by *t-test* using Prism 9. **** *p* < 0.0001, *** *p* < 0.001, mean ± SE (n = 3).

## Morphological analysis and differential counting of the cultured erythroid cells

Cell morphology was assessed using slides prepared with Cytospin using a cyto-centrifuge (Cytospin 3, Shandon Scientific, Tokyo, Japan) at 80 × g for 7 min followed by Wright-Giemsa staining (Sigma Aldrich). Images of the stained cells were taken using an optical microscope (Nikon Eclipse Ti, Tokyo, Japan).

Proerythroblasts, early and late basophilic erythroblasts, polychromatic erythroblasts, orthochromatic normoblasts, and enucleated RBCs were enumerated by differential counting at 200X, magnification using an optical microscope (Nikon Eclipse Ti, Nikon, Tokyo, Japan) following Wright-Giemsa staining.

### Functional analysis of hemoglobin using oxygen equilibrium curve

Hemox Analyzer (TCS, Medical Products Division, Southampton, PA, Canada) was used to record the oxygen equilibrium curve of RBC-like differentiated cells after 24 days. The operating principle of the Hemox Analyzer entails the use of a dual-wavelength spectrophotometer for the measurement of the optical properties of hemoglobin (Hb) and a Clark electrode for measuring the oxygen partial pressure in millimeters of mercury. Both the *p*50 value and observation of the fine structure of the curve can provide information about the delivery of oxygen to tissues. Normal adult peripheral blood was used as the control.

## Results

### Establishment of hEm-iPSCs

The hEm-iPSC lines were obtained by transfection of endometrium cells from five donors using episomal vectors. Several small colonies of approximately 100–200 µm in size were obtained after 14–20 days of transfection and used for differentiation. The stemness of all generated hEm-iPSCs was confirmed as previously described [3]. The established hEm-iPSCs displayed the hallmark characteristics of pluripotency after undirected differentiation via embryoid body (EB) formation. By real-time PCR, the mesoderm markers brachyury, GSC, MIXL1, the endoderm marker sox17, and the ectoderm marker nestin were all clearly expressed, although at different levels when compared with the embryonic stem cell line H9 as the control (Supplementary Fig. s1). For directed differentiation into the hematopoietic lineage, hEm-iPSCs of 15–30 passages were used so that the epigenetic memory of the donor cells may be minimized.

### Hematopoietic differentiation of hEm-iPSCs

In the first phase of hematopoietic differentiation, EB formation was optimized for both hEm-iPSCs, hPB-iPSCs and H9 cells, plated at 1 × 10^6^ cells/well on the AggreWell™ 6 well to allow morphologically uniform EBs to form. The commitment of hEm-iPSCs, hPB-iPSCs and H9 cells lines into the hematopoietic stem cell (HSC) lineage was achieved by co-culturing on OP9 feeder cells. When these EBs were cocultured on the fully expanded OP9 feeder layer, cells with hematopoietic commitment formed a characteristic cogwheel formation on day 8 of culture (Fig. 1).

**Fig. 2:**
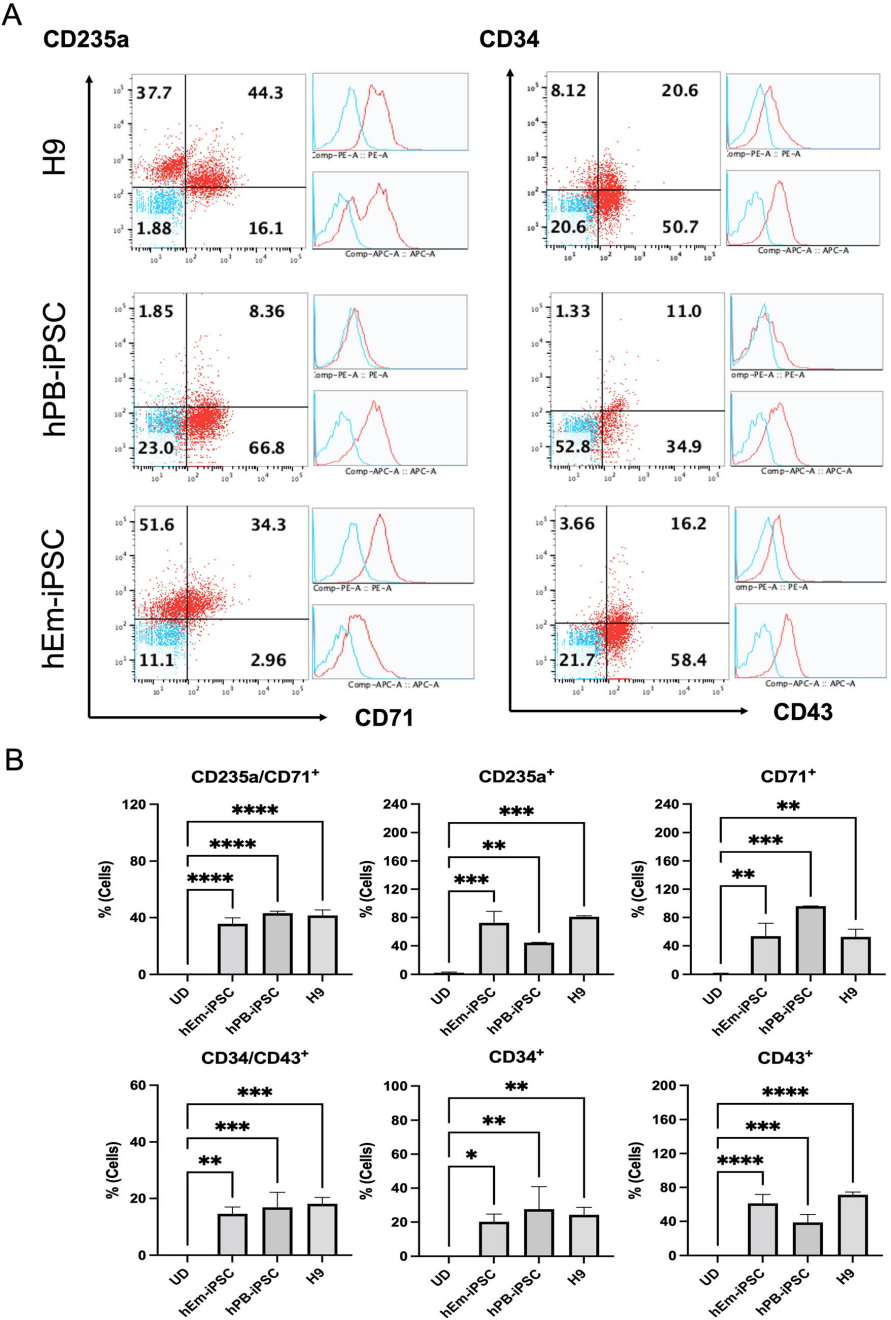
Flowcytometry analysis after hematopoietic expansion. **A** Flowcytometry imaging of cells with hematopoietic commitment after 8 days of co-culture with OP9 feeder cells. **B** The significance of the difference between samples was analyzed by one-way ANOVA using Prism 9. ****p < 0.0001, ***p < 0.001, **p < 0.01, and *p < 0.05, mean ± SE (n = 3)

The cell population shift after 8 days of coculture was measured using flow cytometry analysis. Using flow cytometry, the markers of HSCs (CD34 and CD43), early erythroid progenitors (CD71), and mature erythroid cells (CD235a) were detected. On day 8 of culture, the percentage of CD43^+^ was 61.2 ± 6.1% for hEm-iPSCs, 38.7 ± 5.4% for hPB-iPSCs and 71.3 ± 1.9% for H9 cells. For CD34^+^, the proportion was 20.2 ± 2.6% for hEm-iPSCs, 27.6 ± 7.7% for hPB-iPSCs and 24.3 ± 1.3% for H9 cells. The population percentage showing CD235a expression was 72.5 ± 11.6% for hEm-iPSCs, 44.8 ± 0.8% for hPB-iPSCs and 81.0 ± 1.0% for H9 cells. CD71^+^ was observed in 53.6 ± 10.5% of hEm-iPSCs, 95.9 ± 0.8% for hPB-iPSCs and 52.7 ± 7.7% of H9 cells. CD235a^+^ CD71^+^ was expressed in 35.6 ± 3.7% of hEm-iPSC, 42.1 ± 0.7% for hPB-iPSCs and 41.6 ± 2.8% of H9 cells, indicating that significant HSC and erythroblast progenitor production had taken place (Fig. 2).

### Erythropietic differentiation from hematopoietic stem cells

HSC and erythroblast progenitors obtained from OP9 coculture were subjected to erythropoiesis in the second phase, using feeder-free conditions. Culture conditions were subdivided into three stages over a course of 24 days (Fig. 1).

As erythropoiesis differentiation progressed, the cell population shift of hEm-iPSC, hPB-iPSC and H9 cells was measured by using flow cytometry analysis to detect CD34 and CD43, CD71, and CD235a. Changes in CD34^+^ and CD43^+^, CD71^+^, and CD235a^+^ cell populations were detected as erythroid differentiation proceeded. Antigen presentation for each cell lines varied, but characteristic features could be found (Supplementary Fig. s2). During erythropoiesis of feeder-free culture, a decrease in the CD34^+^ population and a simultaneous subtle increase in the CD43^+^ population was observed for both hEm-iPSCs, hPB-iPSC and H9 cells. In all cell lines, early HPCs (hematopoietic progenitor cells) expressing CD43 were detected from 8 days onwards [19]. The shift in the cell population from the HSC fate to erythroid commitment occurred more efficiently at an earlier timepoint in the hEm-iPSCs derivatives. hEm-iPSCs and H9 cell lines showed similar amplification curves until day 17, after which the CD34^+^CD43^+^ co-expressing HSC population started to decrease in the hEm-iPSC derivatives at 22 days, whereas in the H9 the population increased.

CD71, which is selectively expressed at high levels not only in erythroid precursors but also in all proliferating cells [19], was expressed in all erythroid precursors and even in HSCs. The mature erythroid cell marker, CD235a was prominently observed after 8 days and reached peak levels on day 10 for both hEm-iPSCs, hPB-iPSCs and H9 cells.

At 22 days, CD235a^+^CD71^−^ population, representing the formation of reticulocytes, increased by 21.2 and 15.7% for hEm-iPSCs and H9 cells. hEm-iPSC also strongly expressed the late erythroid markers when compared with the control H9 cells (Supplementary Fig. s2).

### Analysis of the morphological change following the erythropoietic differentiation of hEm-iPSCs

**Fig. 3:**
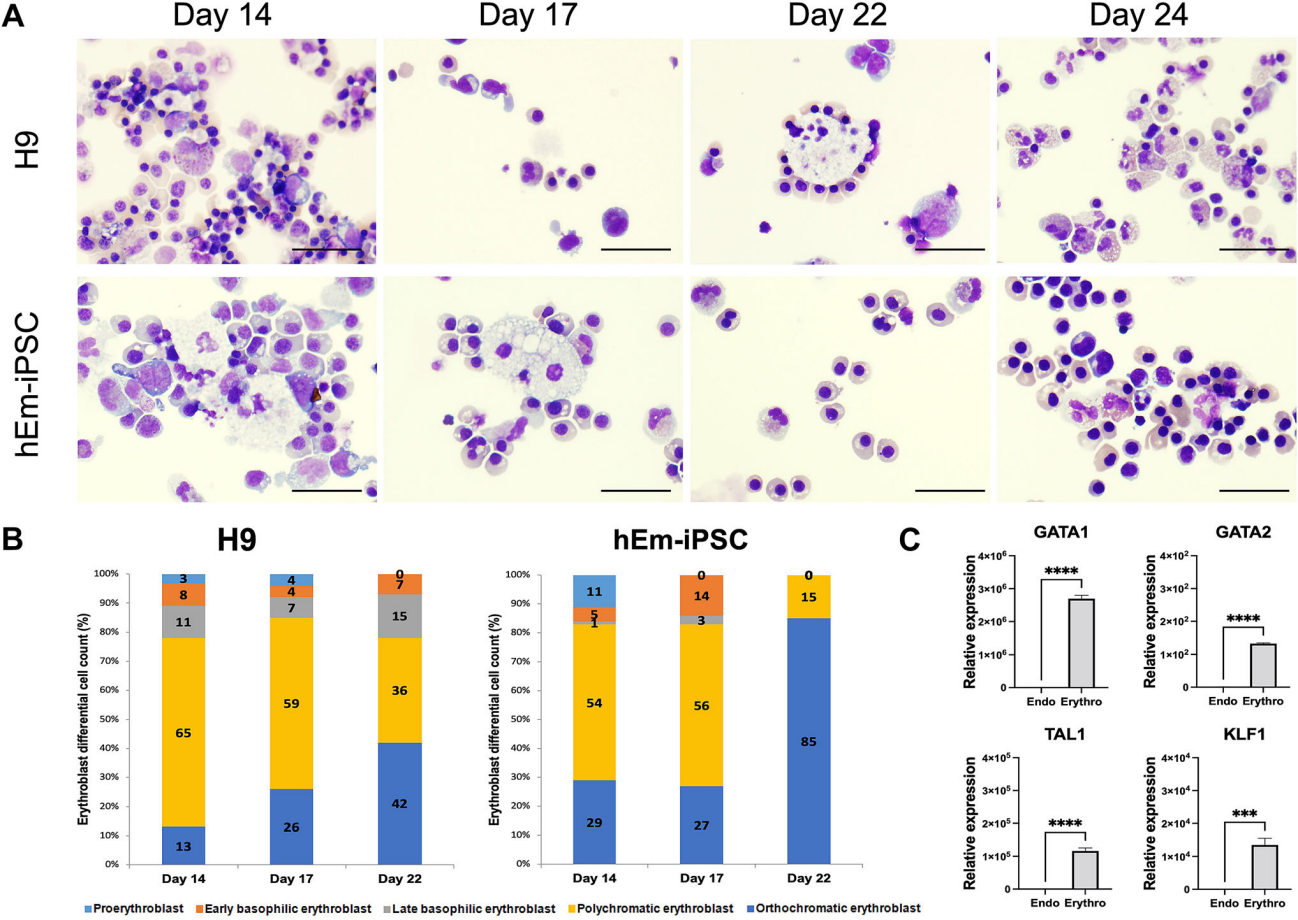
Comparison of cell morphological changes A, cell differential counting B, and expression of functional erythroid regulatory genes C. **A** After Wright-Giemsa staining, erythroid cell types were differentially counted (scale bar presents 20 um). **B** the total pooled proportions of the cells from 3 hEm-iPSCs cultured cells after 14, 17, and 22 days of feeder-free differentiation. **C** GATA1, TAL1, and KLF1 level in hEm-iPSCs (Endo), are increased at day 24 (Erythro) of differentiation. GATA1 is activated and GATA 2 is repressed in late erythroid progenitors (n = 3). The significance of the difference between samples was analyzed by Student’s *t-test* using Prism 9. **** *p* < 0.0001, *** *p* < 0.001, mean ± SE (n = 3)

During the erythropoietic differentiation of *hEm-iPSCs* on days 14, 17, 22, and 24, Wright-Giemsa staining was performed, followed by the differential counting of the cells (Fig. 3A).

On day 14, the most abundant form was polychromatic normoblasts for both hEm-iPSCs and H9 derivatives, the proportions of which were approximately 54 and 65%, respectively. Orthochromatic normoblasts constituted approximately 29% of hEm-iPSCs and 13% of H9 derivatives. On the other hand, the proportion of erythroblasts was 17% (proerythroblasts 11%, early and late basophilic erythroblasts 5 and 1%, respectively) for hEm-iPSCs and 22% (proerythroblasts 3%, early and late basophilic erythroblasts 8 and 11%, respectively) for the H9 derivatives.

On day 17 of feeder-free differentiation, 17% of the hEm-iPSCs were erythroblasts (early and late basophilic erythroblasts, 14 and 3%, respectively) and 15% of the H9 derivatives were erythroblasts (proerythroblasts 4%, early and late basophilic erythroblasts 4 and 7%, respectively). On the other hand, the proportions of normoblasts were 83% for hEm-iPSCs and 85% for the H9 derivatives.

On day 22 of erythropoietic differentiation, the difference in the cell population became more pronounced between the hEm-iPSCs and H9 derivatives. The proportion of erythroblasts at this time point was 0% for hEm-iPSCs and 22% (7% early basophilic and 15% late basophilic erythroblasts) for the H9 derivatives. In contrast, the proportion of normoblasts was 100% for hEm-iPSCs (15% polychromatic normoblasts, 85% orthochromatic normoblasts) and 78% for the H9 derivatives (36% polychromatic normoblasts and 42% orthochromatic normoblasts), indicating a higher differentiation efficiency for hEm-iPSCs (Fig. 3B).

At day 24, the mRNA levels of *GATA1, TAL1*, and *KLF1*, which have key functional roles in erythroid cells, were all strongly expressed in the cell population driven from hEm-iPSCs (Fig. 3C).

### Oxygen binding curves at equilibrium (OEC) by Hemox Analyzer

**Fig. 4:**
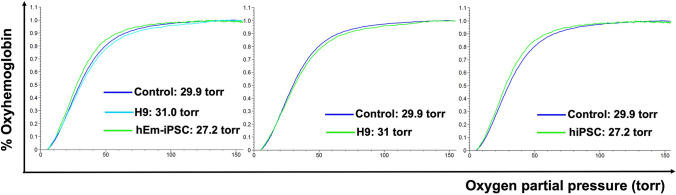
Analysis with Hemox Analyzer in control, H9 and hEm-iPSCs after erythroid differentiation method for 24 days. Differentiated cells were harvested at the end of the 24 days of culture, and oxygen equilibrium was measured by Hemox Analyzer. Control, normal adult peripheral blood; H9, ES cell line differentiated for 24 days, 4.2 × 10^7^ cells; hEm-iPSC, hEm-iPSC line differentiated for 24 days, 3 × 10^7^ cells

According to the Hemox analyzer spectrophotometer, orthochromic normoblasts and mature RBCs derived from hEm-iPS and H9 cell lines showed a similar hemoglobin dissociation curves. These *in vitro* differentiated erythroblasts are able to produce hemoglobin with oxygen binding and dissociation abilities similar to those of normal adult peripheral blood (Fig. 4).

## Discussion

In an era where the average lifespan extends beyond 90 years in many regions of the world, there is an increased demand for improvements in quantity and quality of life. In this context, regenerative medicine is a field of great importance. Awareness of various blood-borne diseases has raised concerns over perioperative transfusions, with no exception to the field of OBGY. Exposure to multiple allogeneic blood transfusions also results in precipitation that induces the formation of irregular antibodies.

Various conditions occurring in women during the reproductive phase frequently involve acute and chronic anemia, and appropriate red blood cell replacement is a vital aspect of conservative and perioperative management. The World Health Organization (WHO) reported that approximately 118.4 million blood donations are collected worldwide in 2018 [27]. Approximately 40% of these are collected in high-income countries accounting for 16% of the world’s population. The risk of transmission of serious infections, including HIV and hepatitis, through unsafe blood transfusions and a worldwide trend of chronic blood shortage has attracted global attention to the safety and availability of usable blood and blood products. With the goal of ensuring universal access to safe blood and blood products, the WHO has issued specific integrated strategies for blood safety and availability. With the increase in blood-borne infectious diseases and aging population in many regions, the number of candidates eligible for blood donation is decreasing. Hence, the discovery of an alternative source of anemia treatment that is safer than allogenic transfusion, and more efficient and faster than iron replacement, is needed.

In the clinical setting, endometrial tissues obtained after hysterectomy could be alternatively obtained through endometrial biopsy, which is minimally invasive and non-scar forming. The hypothesis that innate multipotent cells exist within the endometrium and that its plasticity will serve as an excellent source for reprogramming has been investigated in a few studies. More in-depth studies including directed differentiation using endometrium-driven cell sources are scarce. The merit of inducing erythroid differentiation using recycled post-surgical endometrial tissue, when stored in the form of iPSCs, is that the source is abundant, versatile and could be alternatively used for other on-demand tissue engineering purposes. Moreover, because mature red blood cells are enucleated, differentiated RBCs of iPS cell origin could be a suitable candidate for cell therapy with low concern for ectopic gene integration associated with iPSCs.

Yamanaka et al., who first reported iPSCs derived from murine fibroblasts in 2006 [6], was awarded the Nobel Prize in Medicine only six years after the first description, in recognition of the potential of iPSCs for regenerative medicine and disease modeling. Reprogrammed stem cells are attracting attention not only in the production of next-generation immune-compatible stem cell therapeutics, but also as models for identifying disease etiology, therapeutic efficacy, and toxicity testing [28]. Recently, iPSCs have gained attention as precursor cells for organoid research [29]. An additional advantage of iPSCs is the exemption from ethical issues encountered with embryonic stem cells, while their differentiation potential to different lineages is considered pluripotent.

The idea of having a safer, easily replenished source of red blood cells was made possible with the introduction of iPSCs for the first time [30]. The concept of preserving a self-driven pluripotent cell source that can be indefinitely maintained and expanded has opened numerous possibilities in tissue engineering [31]. Hematopoietic and other blood progenitors with various end-points have been derived from either skin fibroblast-driven iPSCs, cord blood, or peripheral blood mononuclear cells [25, 26, 32–34]. Although the derivation of erythroid lineage cells has been previously attempted by other scholars, the strength of this study lies in the establishment of a full spectrum RBC differentiation protocol from tissues surgically discarded after hysterectomy [2, 3].

In this study, hEm-iPSCs were established from human endometrial cells using episomal iPSC reprogramming vectors (Oct4, Sox2, KFL4, L-Myc, Lin28), which constitutes a transgene-free and virus-free system [11, 12]. hEm-iPSCs were produced and maintained using a feeder-free culture system, and the characteristics of the dedifferentiated stem cell line were confirmed by differentiation into three germ layers. A stepwise strategy involving both feeder and feeder-free conditions was employed for erythroid differentiation to achieve better differentiation efficiency compared to other protocols [35]. Murine bone marrow stromal fibroblasts have been previously utilized to support hematopoietic commitment by providing the appropriate *in vitro* milieu, where the components cannot be fully replaced in feeder-free conditions. Such feeder condition was used in our protocol for robust commitment to hematopoiesis. Consequently, cocktails of specific growth factors could be used to induce *in vitro* hematopoiesis [36], which optimized through this study.

Other groups have attempted various CD34^+^ cell-driven, neural stem cell-driven, and fibroblast-driven iPSCs, claiming that the differentiation efficiency of CD43^+^ cells is similar regardless of donor origin when differentiation is achieved via embryoid body (EB) formation, with efficiencies slightly below 20% [33, 37]. OP9 stromal cells have been reported to enhance the survival of hematopoietic progenitors and precursors derived from human embryonic stem cells (ESCs) and induced pluripotent stem cells (iPSCs) [38, 39]. Therefore, we have adopted the OP9 coculture method in the first phase, as a preliminary, cost-effective and stable approach for erythroid differentiation in our study [37]. Although our system used a shortened 2 day, two phase EB protocol, the rate of CD43^+^ cells using human endometrium-derived iPSCs was between 60 and 70% only after an 8 days of culture, before erythroid commitment was induced. Unlike ES cells, iPSCs are known to possess epigenetic memory of the donor cell; therefore, the efficiency of directed differentiation was reported to be lowered in some studies and not in others [40, 41]. Such impairment in differentiation becomes attenuated as the iPSCs are passed beyond passage 15 [40–43], a characteristic that provides hindrance with regards to time and efficiency. Cells between 20 and 30 passages for hEm-iPSCs, and between 42 and 50 for H9 were used for the differentiation process in the current study.

According to the FACs analysis and Giemsa differential counting, the conversion from hematopoietic stem cells to the erythroid fate is more efficient in the hEm-iPSc compared to the H9 control, the mechanisms of which warrants further analysis. Moreover, the cell source originates from tissue which would otherwise be discarded after pathologic examination, is abundant and easily propagated *in vitro*.

The hallmark of a successful erythroid differentiation is the confirmation of the *in vitro* oxygen-carrying capacity of the final product, which shows that the differentiated erythroblasts can produce hemoglobin with oxygen binding and dissociation abilities similar to that of peripheral blood. Further studies required as an extension of this study is to polish the enucleation process of the normoblasts, and the other blood components including white blood cells and platelet formation could be attempted to be used in tailored *in vitro* disease modelling. In this study, the enucleation rate, which is the possible stage of blood transfusion, was below 10%, and the enucleation of mature RBCs from iPSCs and ESCs has not yet been resolved [44]. Primitive tissues that appear in early embryogenesis are the most suitable preliminary targets for iPS or ES cell differentiation in the field of regenerative medicine, as the signals and environment involved in more complex terminally differentiated tissues are still poorly understood and require further studies.

The present study demonstrates that endometrial cell-derived iPSCs could serve as a reliable source for hematopoietic and erythropoietic differentiation. The current detailed protocol will provide a reproducible and a revisable platform for *in vitro* RBC production as a source of autologous transfusion and for other strategies in regenerative medicine using recycled tissue byproducts in the field of obstetrics and gynecology.

### Supplementary Information

Below is the link to the electronic supplementary material.

Supplemental figure 1. Morphology of generated hEm-iPSCs (40×) and real-time PCR verification of mesoderm (Brachyury, GSC, MIXL1), endoderm (sox17), ectoderm (nestin) germ layer markers. The significance of the difference between samples was confirmed using the Student’s t-test, *p<0.05, **p<0.01, ***p<0.001, mean ± SE. (n=3)

Supplemental figure 2. Flow cytometry analysis of erythroid lineage cells differentiated from hematopoietic stem cells after co-culture with EB on OP9 feeder cells at different stages of the differentiation protocols at days 10, 17, and 22 for hEm-iPSC, hPB-iPSCs and H9 cells (n=3)
