# Supplementary figures and images for: Human Endometrium Derived Induced Pluripotent Stem Cells Are Amenable to Directed Erythroid Differentiation
Source: Tissue Eng Regen Med. 2023 Jul 15;20(6):939–50. doi: 10.1007/s13770-023-00554-9 (PMC10519893; doi:10.1007/s13770-023-00554-9)

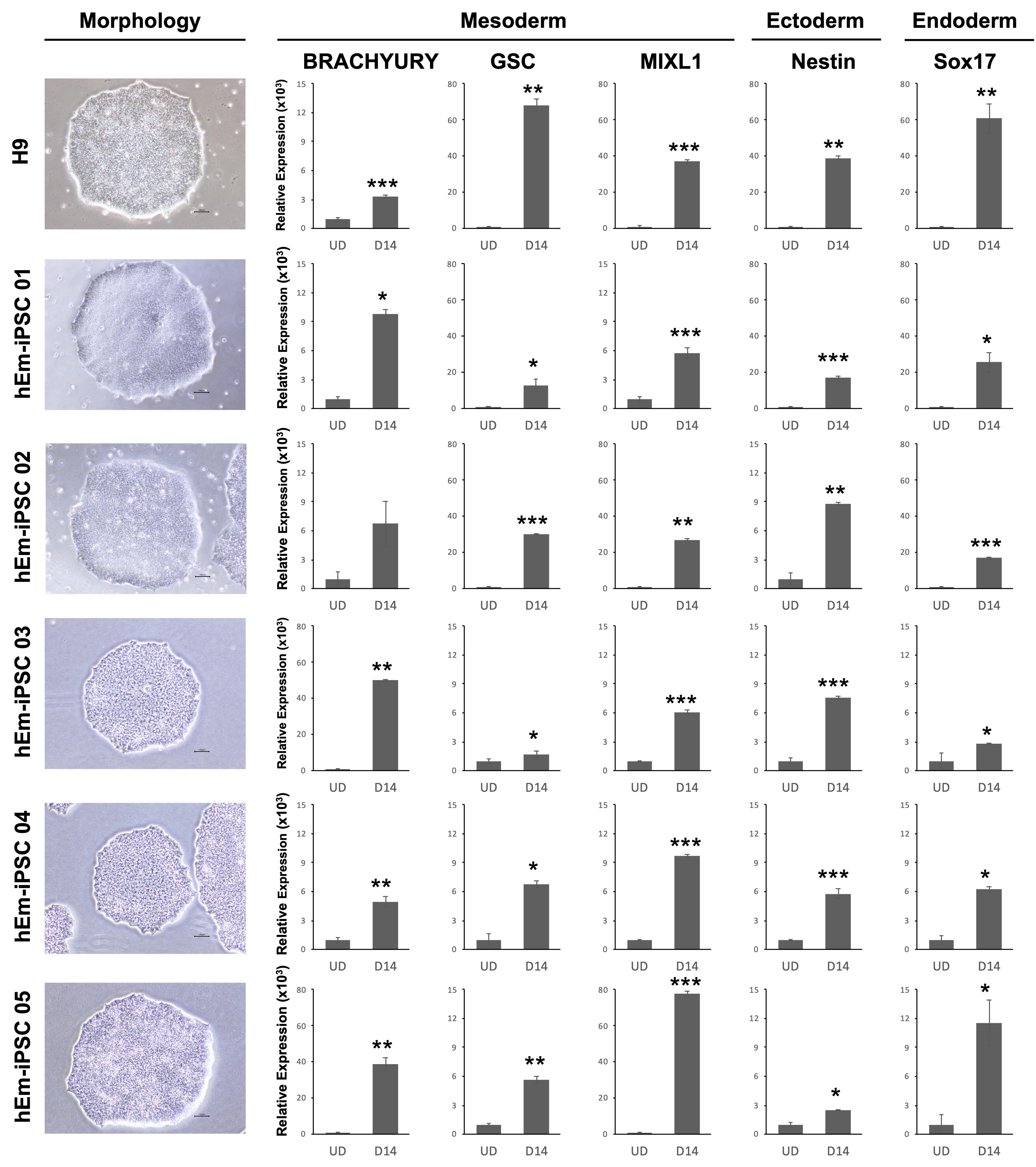

Supplement: Supplementary file 1 — Supplemental figure 1. Morphology of generated hEm-iPSCs (40×) and real-time PCR verification of mesoderm (Brachyury, GSC, MIXL1), endoderm (sox17), ectoderm (nestin) germ layer markers. The significance of the difference between samples was confirmed using the Student’s t-test, *p<0.05, **p<0.01, ***p<0.001, mean ± SE. (n=3) [file 13770_2023_554_MOESM1_ESM.jpg]

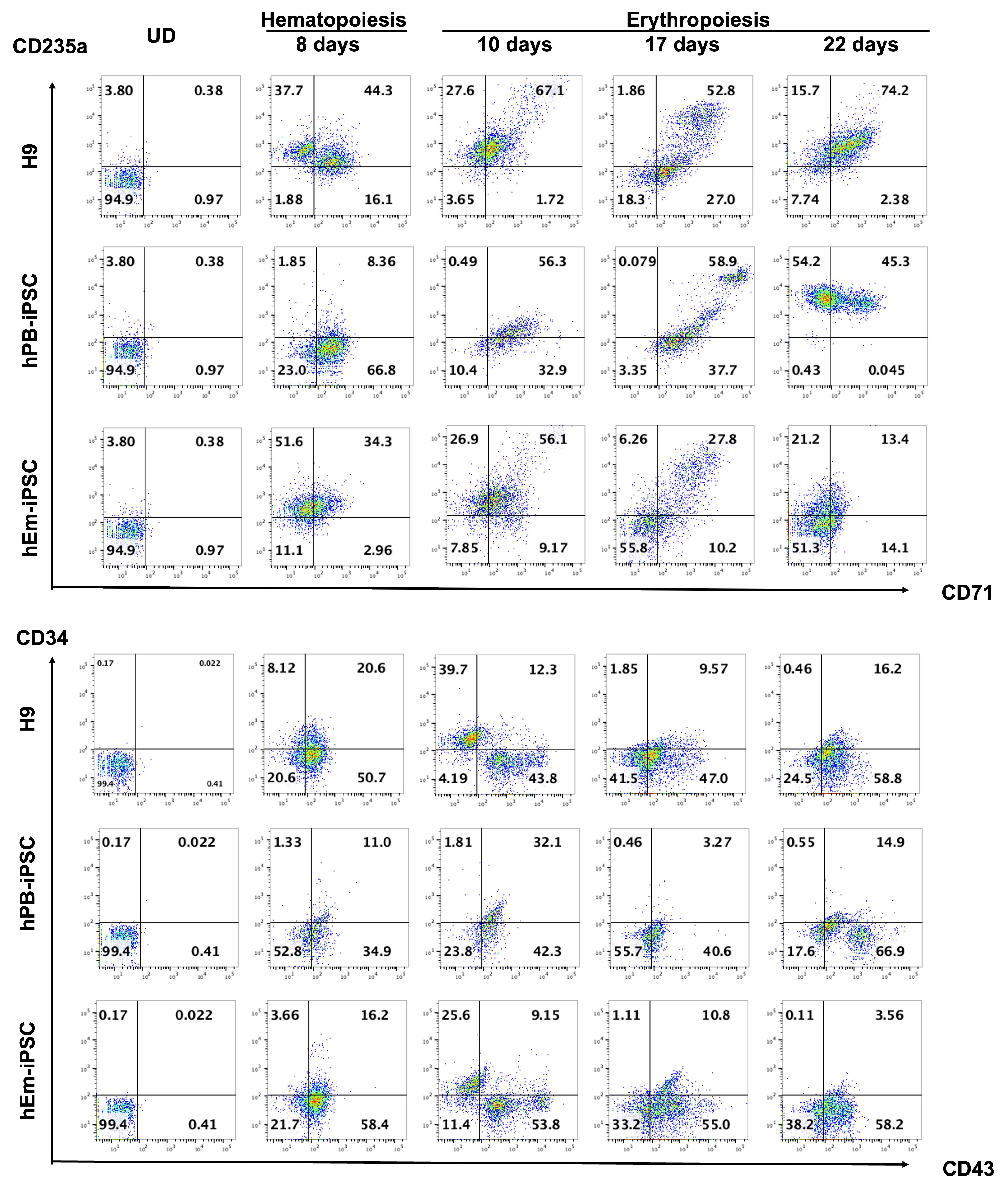

Supplement: Supplementary file 2 — Supplemental figure 2. Flow cytometry analysis of erythroid lineage cells differentiated from hematopoietic stem cells after co-culture with EB on OP9 feeder cells at different stages of the differentiation protocols at days 10, 17, and 22 for hEm-iPSC, hPB-iPSCs and H9 cells (n=3) [file 13770_2023_554_MOESM2_ESM.jpg]
